# Brazilian Organic Honey from Atlantic Rainforest Decreases Inflammatory Process in Mice

**DOI:** 10.3390/vetsci9060268

**Published:** 2022-06-02

**Authors:** Diego Romário-Silva, Josy Goldoni Lazarini, Marcelo Franchin, Severino Matias de Alencar, Pedro Luiz Rosalen

**Affiliations:** 1 Department of Biosciences, Piracicaba Dental School, University of Campinas (UNICAMP), Piracicaba 13414-903, SP, Brazil; d193048@dac.unicamp.br (D.R.-S.); j153162@dac.unicamp.br (J.G.L.); marcelo.franchin@unifal-mg.edu.br (M.F.); 2 Graduate Program in Integrated Dental Sciences, School of Dentistry of the University of Cuiabá, Cuiabá 78065-900, MT, Brazil; 3 Faculty of Dentistry, Federal University of Alfenas, Alfenas 37130-001, MG, Brazil; 4 Department of Agri-Food Industry, Food and Nutrition, “Luiz de Queiroz” College of Agriculture, University of São Paulo (USP), Piracicaba 13418-900, SP, Brazil; smalencar@usp.br; 5 Biological Sciences Graduate Program, Federal University of Alfenas, Alfenas 37130-001, MG, Brazil

**Keywords:** anti-inflammatory, neutrophil migration, cytokine, NF-kB, TNF-α

## Abstract

Honey is an ancient food in the human diet, and the chemical composition of some types of honey has been associated with several beneficial biological effects. Among them, honey has been highlighted to improve health and control inflammatory processes. However, there is no study elucidating the mechanism of action of honey produced organically. Here, we separated organic honey (OH) samples from the Brazilian Atlantic Rainforest into eight different profiles (OH-1 to OH-8) and evaluated, in vitro and in vivo, their anti-inflammatory potential. To determine cell viability, RAW 264.7 macrophages were treated with several concentrations of OH-1 up to OH-8, and anti-inflammatory activity was assessed through NF-κB activation and TNF-α levels. All types of the studied honey up to a concentration of 4% (*w*/*v*) did not interfere with macrophage viability and decreased NF-kB activation and TNF-α levels in macrophage culture in vitro. OH-7 was selected as the most promising anti-inflammatory and used in subsequent assays. Mice pretreated orally with OH-7 showed a decrease in neutrophil migration and TNF-α level. Thus, these types of Brazilian organic honey show promising anti-inflammatory potential, particularly the OH-7 variety. Brazilian organic honey may lead to the development of new products and/or be incorporated into food for use in veterinary medicine and human health as well.

## 1. Introduction

The chemical composition of honey varies according to local flora visited by bees and also the effect of seasonality. In general, honey is a mixture of sugar such as glucose, fructose, sucrose, minerals, vitamins, enzymes, amino acids, organic acids, and phenolic compounds. This complex composition of honey is directly reflected by its biological activities. For years, it has been reported that honey has relevant biological activities such as antimicrobial, anticancer, tissue repair, antioxidant, and anti-inflammatory [1]. Several types of honey from different countries have been associated with promising anti-inflammatory activity, and this effect is associated with a significant amount of phenolic compounds such as quercetin, ellagic acid, gallic acid, caffeic acid, p-coumaric, chrysin, and ferulic acid [2,3,4,5].

The chemical compounds cited above have the capacity to decrease the inflammatory process by controlling inflammatory mediators such as Tumor Necrosis Factor alpha (TNF-α), monocyte chemoattractant protein-1 (MCH-1), and others [6,7,8]. Some diseases related to human beings and veterinary medicine fields, such as arthritis, asthma, cancer, neurodegenerative diseases, inflammatory bowel disease (IBD), chronic inflammatory enteropathies (CIE), and pancreatitis, are an example of pathologies related to uncontrolled and exacerbated inflammatory response [7,8,9].

In the inflammatory process, several mediators are released by immune cells, and in recent years, nuclear factor kappa-B (NF-kB) has been extensively studied due to performing an important role in the inflammatory response, acting on the regulation and expression of inflammatory genes, e.g., cytokines [10]. Hence, the inhibition or regulation of this nuclear factor or cytokines by a natural product, e.g., honey, is an interesting strategy to develop new medicines and products that prevent inflammatory-related diseases.

Thus, some types of honey have been considered a valuable source of compounds that can control the inflammatory process and promising for use in diverse areas such as veterinary, medical products, foods, and others [11,12,13,14]. In a previous study conducted by our research group using organic honey samples, we identified compounds such as ferulic acid, caffeic acid, rutin, and hesperidin. The samples showed antioxidant activity by scavenging ROS/RNS [15]. In the context of organic products, studies show the highest quantity of antioxidant compounds (vitamins, polyphenols, flavonoids) and minerals when related to organic production. These differences in bioactive compound levels are due to the plant being in adverse environmental conditions without the use of pesticides, resulting in greater production of secondary metabolites [16]. Honey has been reported as a functional food with anti-inflammatory activity, and due to being a natural product, there are no adverse effects when compared with anti-inflammatory medicines [17]. However, there is no study evaluating the anti-inflammatory potential to elucidate the mechanism of action of honey produced organically and propose veterinary use.

Our hypothesis is that the chemical compounds present in several types of organic honey and scavenging ROS/RNS can modulate the inflammatory process in in vitro and in vivo inflammatory models. In order to add value to this natural product produced by bees organically, we evaluated, in vitro and in vivo, the anti-inflammatory potential of eight types of organic honey (OH-1 to OH-8) from the Brazilian Atlantic Rainforest.

## 2. Materials and Methods

### 2.1. Reagents

The following reagents were used in this study: formic acid (Tedia, Fairfield, OH, USA); purified water (Millipore Milli-Q System SAS, Molsheim, France); acetonitrile, methanol, and ethanol (J.T. Baker, Phillipsburg, NJ, USA); Roswell Park Memorial Institute (RPMI), lipopolysaccharide (LPS) from *Escherichia coli* 0111:B4, DMSO (dimethylsulfoxide), carrageenan, and 3-(4,5-dimethylthiazol-2-yl)-2,5- diphenyltetrazolium bromide (MTT) (Sigma-Aldrich, St. Louis, MO, USA); fetal bovine serum (FBS) and penicillin/streptomycin (Gibco, Grand Island, NE, USA); RAW 264.7 macrophages transfected with the NF-kB-pLUC gene (Applied Biological Materials Inc., Richmond, BC, Canada); luciferin (Promega Corporation, Madison, WI, USA); TNT lysis buffer and mixture of TRIS and Tween 20 (Amresco, Inc., West Chester, PA, USA); TNF-α kit (R&D Systems, Inc., Minneapolis, MN, USA).

### 2.2. Organic Honey Georeferencing, Collection, and Extraction

Eight samples of certified honey produced organically (OH-1 to OH-8) were collected and geographically referenced in a preserved Brazilian Atlantic Rainforest region from South Paraná State, in the city of General Carneiro (26°25′ S, 51°19′ W) and the city Turvo (25°2′ S, 51°32′ W), between December 2015 and February 2016. The access to Brazilian genetic heritage was requested in accordance with the Brazilian legislation SECEX/CGEN ordinance number 1 and registered on the SISGEN platform under accession number AE0DBB2). All organic honey (OH) samples were diluted to several concentrations using saline or a culture medium and sterilized by filtration.

### 2.3. Honey Profile by High-Performance Liquid Chromatography (HPLC)

Chemical characterization of honey samples (OH-1 to OH-8) was performed using a reversed-phase HPLC instrument equipped with a Shimadzu ODS-A column (RP-18, column size 4.6 × 250 mm; particle size 5 μm) and a DAD detector (SPD-M10AVp, Shimadzu Co., Ltd., Kyoto, Japan). HPLC separation was performed with a Phenomenex Luna C18 column (RP-18, column size 4.6 × 250 mm; particle size 5 µm).

Honey extracts were filtered using a 0.22 µm diameter prior to injection in the HPLC system. The column was eluted using a gradient of water/formic acid (99.75/0.25, *v*/*v*) (solvent A) and acetonitrile/water/formic acid (59.75/40.00/0.25, *v*/*v*/*v*) (solvent B) at a constant flow of 1 mL/min. The gradient of elution was: 10% B, increasing to 20% B (10 min), 30% B (20 min), 50% B (35 min), 50% B (32 min), 90% B (38 min), 90% B (40 min), decreasing to 10% B (45 min). Chromatograms were recorded at 260 nm, as previously described by [15].

### 2.4. In Vitro Anti-Inflammatory Activity

#### 2.4.1. Cell Culture and Viability (MTT)

RAW 264.7 macrophages transfected with the NF-κB-pLUC gene to express luciferase (CQB Number: 022/97) were cultured in endotoxin-free Roswell Park Memorial Institute (RPMI 1640) medium supplemented with 10% fetal bovine serum (FBS) plus 100 U/mL penicillin, 100 μg/mL streptomycin sulfate, and L-glutamine (37 °C, 5% CO_2_). To determine the cytotoxic effects of OH samples, macrophages were cultured in 96-well plates (5 × 10^5^ cells/mL) and incubated for 24 h prior to treatment with OH-1 to OH-8 at 1, 2, 4, and 6% (*w*/*v*) or culture media (negative control). After this period, the supernatant was removed, and the MTT solution (0.3 mg/mL) was added to the wells. The plates remained incubated for 3 h (37 °C, 5% CO_2_). The supernatant was removed, and DMSO 100% (*v*/*v*) was added. Absorbance was measured at 570 nm using an ELISA microplate reader [18]. Triplicate experiments were performed at three independent times.

#### 2.4.2. NF-κB Activation and TNF-α Levels

Transfected macrophage cells were cultured in 24-well plates (3 × 10^5^ cells/mL) overnight. The cells were treated with OH-1 to OH-8 at 0.5, 1, 2, and 4% (*w*/*v*) for 30 min before LPS stimulation (10 ng/mL) for 4 h. The culture media control was used as a negative control. After 4 h, cell lysis buffer and 25 μL of luciferase reagent (luciferin at 0.5 mg/mL) were added to each well. Luminescence was measured using a white microplate reader (SpectraMax M3, Molecular Devices). For the measurement of TNF-α levels, the supernatant was recovered and determined according to the protocols provided by the manufacturers (R&D Systems, Minneapolis, MN, USA) and determined using an ELISA microplate reader. The results were expressed as pg/mL [19]. Triplicate experiments were performed at three independent times.

### 2.5. In Vivo Anti-Inflammatory Study

#### 2.5.1. Animals

To determine the capacity of OH to decrease neutrophil influx into the inflamed site, male specific-pathogen-free (SPF) C57BL/6Junib mice, purchased from CEMIB/UNICAMP (Multidisciplinary Center for Biological Research, Campinas, SP, Brazil), weighing between 22 and 25 g, were used. All animals were housed in a vivarium under humidity (40–60%) and temperature (22 ± 2 °C) control in 12 h light–dark cycle, with access to food and water ad libitum. For the experiment, all animals were deprived of food for 8 h before oral administration. This study complied with the National Council for Animal Experimentation Control guidelines for the care and use of animals in scientific experimentation (NIH Publication Number: 85-23, revised in 1985). The study protocol was previously approved by the Institutional Ethics Committee on Animal Research at the University of Campinas (CEUA/UNICAMP, Protocol Number: 5348-1).

#### 2.5.2. Neutrophil Migration into the Peritoneal Cavity of Mice and TNF-α Levels

Mice were pretreated orally (via gavage) five times a day for five days with 100 μL of OH-7 at 40% and 100 μL of OH-7 in natura (100%). The negative control group received oral administration of 0.9% saline (vehicle). After five days, all animals received an inflammatory challenge by intraperitoneal (i.p.) injection of the carrageenan (500 μg/cavity) in the peritoneum for 4 h, except the vehicle group. After 4 h of challenge, mice were euthanized, and their peritoneal cavity was washed and recovered to count the total number of leukocytes and neutrophils. The volumes recovered were similar in all experimental groups and equated to approximately 95% of the injected volume. The results were expressed as the number of neutrophils per cavity. To measure the release of TNF-α levels, peritoneal fluid was recovered and determined according to the protocols provided by the manufacturers (R&D Systems, Minneapolis, MN, USA) and determined using an ELISA microplate reader. The results were expressed as pg/mL [19].

### 2.6. Statistical Analysis

The data were checked for normality and submitted to one-way analysis of variance (ANOVA), followed by Tukey’s post-hoc test. The results were expressed as mean ± standard deviation (SD). The results were considered significant at *p* < 0.05 and α: 5%.

## 3. Results

### 3.1. Organic Honey Characterization

The collected organic honey samples are in accordance with our previous work [15], where organic blossom honey and honeydew honey from southern Brazil were separated into eight different profiles (OH-1 to OH-8).

### 3.2. In Vitro Anti-Inflammatory Activity

#### 3.2.1. Cell Viability Assay

**Figure 1:**
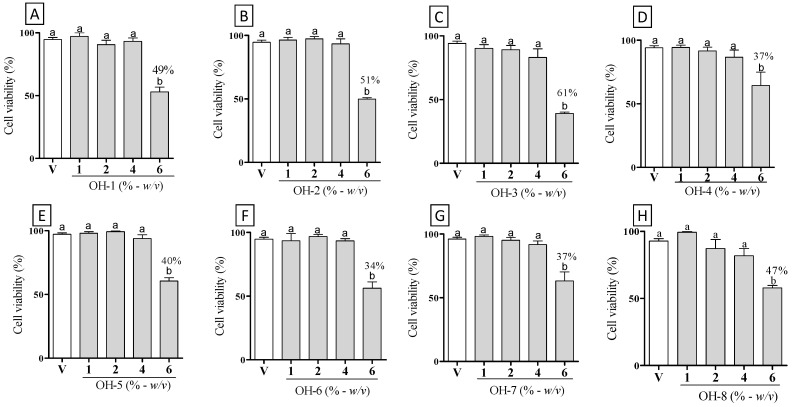
Cell viability with assay MTT. RAW 264.7 macrophages were treated with vehicle-RPMI medium (V) and different concentrations (% - *w*/*v*) of Brazilian organic honey: (**A**) OH-1, (**B**) OH-2, (**C**) OH-3, (**D**) OH-4, (**E**) OH-5, (**F**) OH-6, (**G**) OH-7, and (**H**) OH-8. Different letters indicate statistically significant intragroup differences (*p* < 0.05; one-way ANOVA, followed by Tukey’s post-hoc test).

As seen in Figure 1, the macrophages pretreated with honey did not significantly affect cell viability up to 4% (Figure 1A–H). However, all samples induced significantly cell cytotoxicity at 6% as compared to the culture media control (*p* < 0.05).

#### 3.2.2. NF-κB Activation and TNF-α Levels

**Figure 2:**
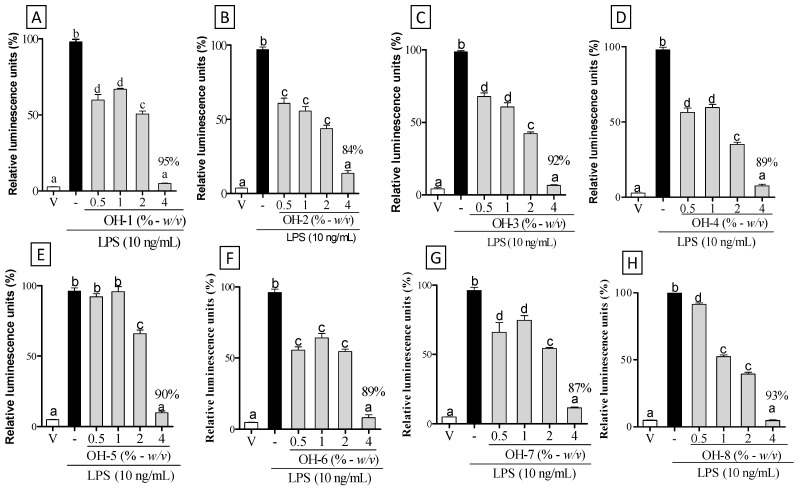
Anti-inflammatory potential of organic honey. To evaluate NF-κB activation, macrophages were treated with vehicle—V (RPMI medium) and LPS (-, 10 ng/mL): (**A**) OH-1, (**B**) OH-2, (**C**) OH-3, (**D**) OH-4, (**E**) OH-5, (**F**) OH-6, (**G**) OH-7, and (**H**) OH-8 in different concentrations (% - *w*/*v*) for 30 min before LPS stimulation. The negative control group received 0.9% saline (vehicle—V). Different letters indicate statistically significant intragroup differences (*p* < 0.05; one-way ANOVA, followed by Tukey’s post-hoc test).

To verify the anti-inflammatory potential of the OH, we carried out NF-κB activation in macrophages. As seen in Figure 2, macrophages treated with the highest concentration (4%) of OH-1 to OH-8 showed decreased NF-κB activation (95%, 84%, 92%, 89%, 90%, 89%, 87%, and 93%, respectively) as compared to their respective LPS group (Figure 2A–H; *p* < 0.05). Treatment with OH-1 to OH-4 and OH-6 to OH-8 at 0.5%, 1%, and 2% also decreased NF-κB activation as compared to their respective LPS group (Figure 2A–D,F–H; *p* < 0.05). Macrophages treated with OH-5 at 2% showed decreased NF-κB activation as compared to the LPS group; however, the 0.5 and 1% OH-5 treatment did not reduce NF-κB activation (*p* > 0.05).

**Figure 3:**
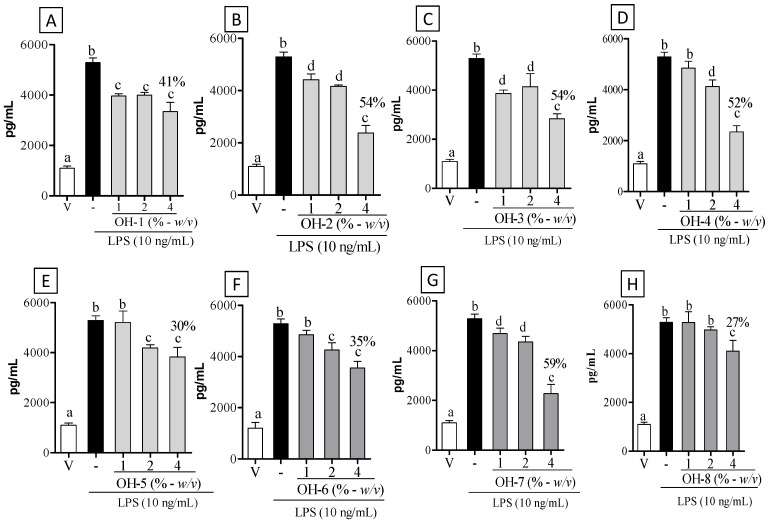
Effect of organic honey (OH-1 to OH-8) in decreasing the release of TNF-α in RAW 264.7 macrophages. Macrophages were treated with vehicle—V (RPMI medium) and LPS (-, 10 ng/mL): (**A**) OH-1, (**B**) OH-2, (**C**) OH-3, (**D**) OH-4, (**E**) OH-5, (**F**) OH-6, (**G**), OH-7, and (**H**) OH-8 in different concentrations for 30 min before LPS stimulation. The negative control group received 0.9% saline (vehicle—V). Different letters indicate statistically significant intragroup differences (*p* < 0.05; one-way ANOVA, followed by Tukey’s post-hoc test).

OH-1 to OH-8 were tested for their potential to decrease the proinflammatory cytokine TNF-α. As seen in Figure 3, macrophages pretreated with OH-1 to OH-8 at 4% showed a significant decrease in TNF-α level (41%, 54%, 54%, 52%, 30%, 35%, 59%, and 27%, respectively) as compared to their LPS group (Figure 3A–H; *p* < 0.05). OH-1, 2, 3 and 7 at 1% decreased TNF-α level as compared to their LPS group (Figure 3A–C,G; *p* < 0.05). Lastly, the treatment with OH-1 to OH-7 at 2% decreased TNF-α level as compared to their LPS group (Figure 3A–G; *p* < 0.05).

### 3.3. In Vivo Anti-Inflammatory Study

#### Neutrophil Migration into the Peritoneal Cavity of Mice and TNF-α Levels

**Figure 4:**
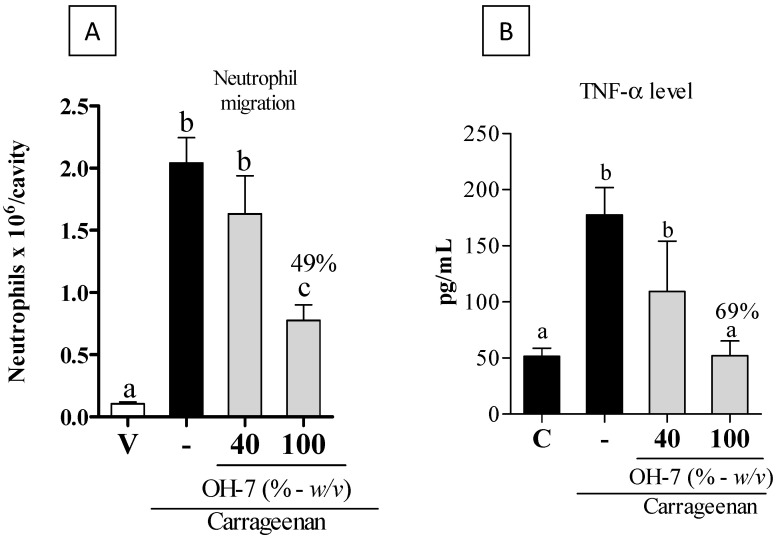
Effects of OH-7 on neutrophil migration and TNF-α release in vivo. (**A**) Effect of vehicle—V (NaCl solution 0.9%), carrageenan (-), and OH-7 (40% and 100%) on neutrophil migration into the peritoneal cavity of mice induced by i.p. administration of carrageenan (500 μg/cavity-). (**B**) Effect of the OH-7 treatment on the release of TNF-α in mice. The results were expressed as mean ± SD, *n* = 5–6. Different letters indicate statistically significant intragroup differences (*p* < 0.05; One-way ANOVA, followed by Tukey’s post-test).

In order to define the most promising sample among all, OH-7 was found to be the most bioactive, and then it was followed with a preclinical anti-inflammatory assay using an animal model. Then, the inhibitory effects of OH-7 on neutrophil influx were determined in vivo. As seen in Figure 4A, mice pretreated with OH-7 at 100% (in natura) showed a significant decrease in neutrophil influx (49%) as compared to those treated with the control carrageenan (*p* < 0.05). However, OH-7 at 40% did not decrease neutrophil migration (*p* > 0.05). Regarding the release of TNF-α levels, mice pretreated with OH-7 at 100% (in natura) showed a significant decrease in TNF-α level (69%) as compared to those treated with the control carrageenan (Figure 4B). However, OH-7 at 40% did not decrease TNF-α level (*p* > 0.05).

## 4. Discussion

In veterinary medicine, some diseases are diagnosed as immunologically mediated such as canine and feline IBD, chronic inflammatory enteropathies (CIE), pancreatitis, and ovarian dysfunction in cows, leading to infertility (uterine inflammatory diseases), and others are related to uncontrolled inflammation [7,8,9]. Honey, a natural animal product produced by bees, especially the *Apis mellifera* species, has been observed to control human and animal diseases related to the inflammatory process by decreasing biomarkers such as proinflammatory cytokines and inflammation-related enzymes [20,21,22].

Despite being an animal product, the chemical composition of honey varies according to the flora visited by bees, as well as seasonality. In general, honey is a mixture of sugar, carbohydrates, minerals, vitamins, enzymes, amino acids, organic acids, alkaloids, and phenolic compounds [23]. In addition, honey has been shown to have an enzymatic complex composed of diastase, invertases, glucose oxidase, catalase, and acid phosphatase [24].

The chemical complexity of honey reflects several biological activities that may modulate biological functions. Thus, honey is a functional food that supplies nutritional needs and can prevent the occurrence of inflammatory diseases [25]. Despite all the benefits of honey [1], the anti-inflammatory activity of certified honey produced organically has not been found in the scientific literature. Thus, it is justifiable to carry out research with honey produced organically in order to elucidate possible differences between the biological activities reported in conventional honey. The production of organic honey has important ecological concepts, and for this purpose, the use of good agricultural practices is indispensable to maintain the balance and diversity of the ecosystem where honey is produced. Thus, It is possible to promote the sustainable use of natural resources, environmental quality, animal welfare, and human health [26]. Several criteria characterize the production of bee products as organic, including pollen collection areas with adequate nutrition, preventive bee health measures, areas free of human activity, use of fertilizers, and use of bees without genetic modification [27]. In our study, the samples of honey were classified by HPLC chemical profile into eight types of organic blossom honey and honeydew honey from southern Brazil. This classification was in accordance with our previous work [15].

Therefore, the anti-inflammatory mechanism of action of the eight types of organic honey (OH-1 to OH-8) from the Brazilian Atlantic Rainforest was evaluated.

Anti-inflammatory assays of organic honey were conducted by macrophage cell culture, evaluating the reduction of NF-κB activation and release of TNF-α levels. Firstly, the cell viability assay was conducted to evaluate whether organic honey showed cytotoxicity, despite honey being a nontoxic food to human beings. None of the organic honey samples showed cell toxicity up to 4% (*w*/*v*); however, at the highest concentration (6%), all samples induced toxicity, which can be explained by the osmosis change provided by honey becoming a hypertonic solution to the macrophages. Regarding anti-inflammatory activity, all samples of organic honey at 4% decreased NF-κB activation, and these preliminary results indicated promising anti-inflammatory activity since NF-κB is involved in different intracellular inflammatory pathways. NF-κB represents a nuclear transcription family that regulates a wide amount of genes involved in the immunological system, including the inflammatory process of the organism [28]. Since immune system cells, such as macrophages, are bound by a molecule, e.g., lipopolysaccharide (LPS), this nuclear transcription family is triggered and initiates cascade-regulating processes such as differentiation of T lymphocytes, release of cytokines, pattern recognition receptors (PRRs), and others [29,30]. Thus, the deregulation of NF-κB is intrinsically related to the appearance and progression of chronic inflammatory diseases, and the therapeutic approach that may interfere in a controlled manner with exacerbated NF-κB activation represents an important strategy that must be explored and developed [31].

Several cells of the immune system, including macrophages, have intracellular NF-κB that can be activated by canonical and noncanonical (alternative) pathways. The canonical pathway is related to the signaling of TNF-α and IL-1, proinflammatory cytokines in the context of chronic inflammatory diseases [32]. After the inflammatory cells release these mediators, they initiate protein expression under the endothelial cell surface, such as selectins and integrins, which increase and direct the flow of neutrophils to the inflammatory focus for diapedesis [32]. In this sense, some inflammatory diseases are related to veterinary medicine fields, such as mastitis, inflammatory bowel disease, and ruminal acidosis in cows, and others are related to disordered NF-κB activation [33,34,35].

The intrinsic relationship between NF-κB activation and TNF-α levels is well known [36]. To verify whether organic honey decreases inflammatory parameters in vitro, NF-κB activation and release of TNF-α levels were carried out. After 4 h of honey treatment in macrophages, a decrease in NF-κB activation and release of TNF-α levels by macrophages treated with OH-7 was observed, showing a percentage of inhibition of 85% and 59%, respectively. In a study conducted by Minden-Birkenmaier and Meadows in 2019 [37], the anti-inflammatory activity of Manuka honey and medical-grade honey from New Zealand was evaluated in HL-60 cells treated with 0.5% and 3% (*v*/*v*). They concluded that Manuka honey at 0.5% reduced the release of TNF-α levels and other inflammatory mediators; however, cells treated at 3% showed an increase in TNF-α release level [37]. In contrast, the eight samples of Brazilian organic honey decreased the release of TNF-α at 4% (*w*/*v*). This result may be explained due to variations related to the chemical and enzymatic profile of organic honey and the different cell lines used in both studies compared as well. It is worth noting that in both studies, honey represents an important source to reduce inflammatory mediators in chronic diseases.

Collectively, among all the samples analyzed, OH-7 was the most promising to decrease inflammatory markers such as NF-κB and TNF-α; thus, it was selected for the subsequent in vivo anti-inflammatory assay. Mice pretreated with OH-7 before challenge by carrageenan-induced peritonitis showed a decrease in neutrophil influx (49%) compared to the control. Similarly, the release of TNF-α levels by mice decreased (69%). These findings indicate that OH-7 has promising anti-inflammatory potential for acute inflammation. Studies evaluating chronic inflammatory conditions must be conducted to select honey as a functional food (adjuvant therapy) in chronic diseases. Furthermore, our findings validate the hypothesis that organic honey consumption decreases leukocyte migration and, consequently, the release of inflammatory mediators. A research group evaluated the in vivo anti-inflammatory effect of Malaysian Gelam honey using a carrageenan-induced paw edema model [38]. The authors found that the animals showed a decrease in paw edema in a dose-dependent manner and a reduction in TNF-α levels in plasma and paw tissue. In another study, the authors evaluated the anti-inflammatory potential of Manuka honey and concluded that at 0.5, 1, and 3%, the sample reduced the phosphorylation of IκBα in a dose-dependent manner, also decreasing chemotaxis and, consequently, the reduction of neutrophil influx. Manuka honey is a medical-grade honey known worldwide for its antimicrobial and anti-inflammatory potential. This honey has been used in the hospital environment in New Zealand to treat inflamed and infected wounds resistant to allopathic drugs [38,39]. Thus, OH-7 showed anti-inflammatory activity similar to Manuka honey, and therefore, it can be considered a promising natural product and candidate for Brazilian medical-grade honey.

Moreover, to stress the importance of honey as a functional food or therapeutic input, e.g., Manuka honey [37], studies that are biologically guided (bioguided) need to be encouraged mainly in the matter of natural products [40]. Honey has a chemical profile that varies according to local flora and reflects secondary metabolites from different plants, increasing the chances of discovering promising molecules with biological activities for the development of medicines [41]. The anti-inflammatory activity of honey has been reported due to its expressive quantity of phenolic composition [42]. Phenolic compounds have been related to decreasing the release of proinflammatory mediators by interfering with enzymes such as inducible nitric oxide synthase (iNOS), cyclooxygenase-1 (COX-1), and cyclooxygenase-2 (COX-2) [42]. Previously, our research group studied the antioxidant activity of these eight organic honey samples (OH-1 to OH-8) and also identified the main chemical compounds of crude honey extracts [15]. The total phenolic content found in honey extracts (mg—GAE/g) was 73.15 (OH-1), 59.79 (OH-2), 49.79 (OH-3), 52.20 (OH-4), 117.68 (OH-5), 84.08 (OH-6), 83.19 (OH-7), and 53.03 (OH-8). In total, four phenolic compounds were identified in the samples such as ferulic acid, caffeic acid, rutin, and hesperidin. In addition, we identified considerable amounts of ascorbic acid, ranging from 2.75 to 6.22 mg/100 g in OH-3, OH-5, and OH-7 [15]. The presence of these compounds can explain, in part, the in vitro anti-inflammatory activity exerted by all types of organic honey and in vivo by OH-7.

In this context, it is important to note that the chemical composition of honey is influenced by propolis composition. Two phenolic acids, ferulic acid and caffeic acid, present in propolis and also organic honey, were related to the decrease in inflammatory markers such as cyclooxygenase (COX-2), iNOS protein expression, and prostaglandin (PGE2) [43,44]. In addition, [45] observed that hesperidin, present in propolis and also organic honey, has immunosuppressive effects, decreasing the release of IL-12, IL-10, IL-4, and IL-2 by peripheral blood mononuclear cells. Despite this, phenolic compounds alone do not explain the biological activities of honey. Studies have shown that some glycoproteins and sugars present in honey may be related to anti-inflammatory activity [46,47,48,49]. Therefore, we suggest that the positive results for the anti-inflammatory activity of the honey presented here are due to a synergistic effect between the honey components. Furthermore, a study has shown that not only the chemical composition of honey but also the architecture of these components within its composition can influence its biological activities [50].

In this sense, propolis has many applications in the veterinary field such as protecting mammary bovine cells against pathogens, acting as an antimicrobial, increasing the digestibility of dry matter in dairy cows’ rumen, promoting cicatrization in horses and dogs, inhibiting the growth of buffalo rumen ciliate protozoa, and others [44]. Considering the success of propolis use in the veterinary medicine field, organic honey from bees is considered a promising source of compounds with anti-inflammatory activity and opens new perspectives concerning its application in veterinary products.

## 5. Conclusions

Brazilian organic honey reduced the inflammatory process through the modulation of NF-κB activation and TNF-α levels, and the OH-7 variety was the most promising. This study adds value to the promising Brazilian organic honey, which is considered a promising source of compounds with anti-inflammatory activity, and opens new perspectives concerning its application in veterinary products, providing benefits for animal health (functional foods), contributing to the development of agribusiness for many families, and also contributing to the preservation of biodiversity, particularly in the Brazilian Atlantic Rainforest.

## Data Availability

Not applicable.

## References

[1] Cooper R. (2016). Honey for wound care in the 21st century. J. Wound Care.

[2] Kassim M., Achoui M., Mustafa M.R., Mohd M.A., Yusoff K.M. (2010). Ellagic acid, phenolic acids, and flavonoids in Malaysian honey extracts demonstrate in vitro anti-inflammatory activity. Nutr. Res..

[3] Hadagali M.D., Chua L.S. (2014). The anti-inflammatory and wound healing properties of honey. Eur. Food Res. Technol..

[4] Hussein S.Z., Mohd Yusoff K., Makpol S., Mohd Yusof Y.A. (2013). Gelam Honey Attenuates Carrageenan-Induced Rat Paw Inflammation via NF-κB Pathway. PLoS ONE.

[5] Ranneh Y., Akim A.M., Hamid H.A., Khazaai H., Fadel A., Zakaria Z.A., Albujja M., Bakar M.F.A. (2021). Honey and its nutritional and anti-inflammatory value. BMC Complement. Med. Ther..

[6] De Melo T.S., Lima P.R., Carvalho K.M., Fontenele T.M., Solon F.R., Tomé A.R., de Lemos T.L., da Cruz Fonseca S.G., Santos F.A., Rao V.S. (2017). Ferulic acid lowers body weight and visceral fat accumulation via modulation of enzymatic, hormonal and inflammatory changes in a mouse model of high-fat diet-induced obesity. Braz. J. Med. Biol. Res..

[7] Magata F. (2020). Lipopolysaccharide-induced mechanisms of ovarian dysfunction in cows with uterine inflammatory diseases. J. Reprod. Dev..

[8] Jergens A.E., Simpson K.W. (2012). Inflammatory bowel disease in veterinary medicine. Front. Biosci..

[9] Heilmann R.M., Steiner J.M. (2018). Clinical utility of currently available biomarkers in inflammatory enteropathies of dogs. J. Vet. Intern. Med..

[10] Henríquez-Olguín C., Altamirano F., Valladares D., López J.R., Allen P.D., Jaimovich E. (2015). Altered ROS production, NF-κB activation and interleukin-6 gene expression induced by electrical stimulation in dystrophic mdx skeletal muscle cells. Biochim. Biophys. Acta Mol. Basis Dis..

[11] Dias D.A., Urban S., Roessner U. (2012). A Historical overview of natural products in drug discovery. Metabolites.

[12] Choy E. (2012). Understanding the dynamics: Pathways involved in the pathogenesis of rheumatoid arthritis. Rheumatology.

[13] El-Seedi H.R., Eid N., Abd El-Wahed A.A., Rateb M.E., Afifi H.S., Algethami A.F., Zhao C., Al Naggar Y., Alsharif S.M., Tahir H.E. (2022). Honey Bee Products: Preclinical and Clinical Studies of Their Anti-inflammatory and Immunomodulatory Properties. Front. Nutr..

[14] Samarghandian S., Farkhondeh T., Samini F. (2017). Honey and health: A review of recent clinical research. Pharmacogn. Res..

[15] Silva C.F., Rosalen P.L., Soares J.C., Massarioli A.P., Campestrini L.H., Semarini R.A., Ikegaki M., Alencar S.M. (2020). Polyphenols in Brazilian organic honey and their scavenging capacity against reactive oxygen and nitrogen species. J. Apic. Res..

[16] Young J.E., Zhao X., Carey E.E., Welti R., Yang S.S., Wang W. (2005). Phytochemical phenolics in organically grown vegetables. Mol. Nutr. Food Res..

[17] Nooh H.Z., Nour-Eldien N.M. (2016). The dual anti-inflammatory and antioxidant activities of natural honey promote cell proliferation and neural regeneration in a rat model of colitis. Acta Histochem..

[18] Denizot F., Lang R. (1986). Rapid colorimetric assay for cell growth and survival. Immunol. Methods.

[19] Lazarini J.G., de Cássia OrlandiSardi J., Franchin M., Nani B.D., Freires I.A., Infante J., Paschoal J.A.R., Alencar S.M., Rosalen P.L. (2018). Bioprospection of *Eugenia brasiliensis*, a Brazilian native fruit, as a source of anti-inflammatory and antibiofilm compounds. Biomed. Pharmacother..

[20] Shivappa N. (2019). Diet and chronic diseases: Is there a mediating effect of inflammation?. Nutrients.

[21] Khan R.U., Naz S., Abudabos A.M. (2017). Towards a better understanding of the therapeutic applications and corresponding mechanisms of action of honey. Environ. Sci. Pollut. Res. Int..

[22] Alqarni A.S., Owayss A.A., Mahmoud A.A., Hannan M.A. (2014). Mineral content and physical properties of local and imported honeys in Saudi Arabia. J. Saudi Chem. Soc..

[23] Escuredo O., Dobre I., Fernández-González M., Seijo M.C. (2014). Contribution of botanical origin and sugar composition of honeys on the crystallization phenomenon. Food Chem..

[24] Estevinho L., Pereira A.P., Moreira L., Dias L.G., Pereira E. (2008). Antioxidant and antimicrobial effects of phenolic compounds extracts of Northeast Portugal honey. Food Chem. Toxicol..

[25] Ozen A.E., Pons A., Tur J.A. (2012). Worldwide consumption of functional foods: A systematic review. Nutr. Rev..

[26] Khalifa S.A.M., Elshafiey E.H., Shetaia A.A., El-Wahed A.A.A., Algethami A.F., Musharraf S.G., AlAjmi M.F., Zhao C., Masry S.H.D., Abdel-Daim M.M. (2021). Overview of Bee Pollination and Its Economic Value for Crop Production. Insects.

[27] Jahn G., Schramm M., Spiller A. (2005). The Reliability of Certification: Quality Labels as a Consumer Policy Tool. J. Consum. Policy.

[28] Oeckinghaus A., Ghosh S. (2009). The NF-κB Family of Transcription Factors and and its regulation. Cold Spring Harb. Perspect. Biol..

[29] Lawrence T. (2009). The nuclear factor NF-kappaB pathway in inflammation. Cold Spring Harb. Perspect. Biol..

[30] Sun S.C., Chang J.H., Jin J. (2013). Regulation of NF-κB in Autoimmunity. Trends Immunol..

[31] Liu T., Zhang L., Joo D., Sun S.C. (2017). NF-κB signaling in inflammation. Signal. Transduct. Target. Ther..

[32] Noort A.R., Tak P.P., Tas S.W. (2015). Non-canonical NF-kB signaling in rheumatoid arthritis: Dr Jekyll and Mr Hyde?. Arthritis Res. Ther..

[33] Guo Y.F., Xu N.N., Sun W., Zhao Y., Li C.Y., Guo M.Y. (2017). Luteolin reduces inflammation in Staphylococcus aureus-induced mastitis by inhibiting NF-kB activation and MMPs expression. Oncotarget.

[34] Kathrani A., Holder A., Catchpole B., Alvarez L., Simpson K., Werling D., Allenspach K. (2012). TLR5 risk-associated haplotype for canine inflammatory bowel disease confers hyper-responsiveness to flagellin. PLoS ONE.

[35] Jin D., Chang G., Zhang K., Guo J., Xu T., Shen X. (2016). Rumen-derived lipopolysaccharide enhances the expression of lingual 485 antimicrobial peptide in mammary glands of dairy cows fed a high-concentrate diet. BMC Vet. Res..

[36] Wong E.T., Tergaonkar V. (2009). Roles of NF-κB in health and disease: Mechanisms and therapeutic potential. Clin. Sci..

[37] Minden-birkenmaier B.A., Meadows M.B. (2019). The Effect of Manuka Honey on dHL-60 Cytokine, Chemokine, and Matrix-Degrading Enzyme Release under Inflammatory Conditions. Med. One.

[38] Hussein S.Z., Mohd Yusoff K., Makpol S., Mohd Yusof Y.A. (2012). Gelam honey inhibits the production of proinflammatory, mediators NO, PGE 2, TNF-α, and IL-6 in carrageenan-induced acute paw edema in rats. Evid. Based Complement. Altern. Med..

[39] Shan Y. (2019). Medicinal honey in clinical practice: Viable alternative or useful adjunct in wound care management?. Br. J. Nurs..

[40] Pieters L., Vlietinck A.J. (2005). Bioguided isolation of pharmacologically active plant components, still a valuable strategy for the finding of new lead compounds?. J. Ethnopharmacol..

[41] Nolan V.C., Harrison J., Cox J.A.G. (2019). Dissecting the antimicrobial composition of honey. Antibiotics.

[42] Masad R.J., Haneefa S.M., Mohamed Y.A., Al-Sbiei A., Bashir G., Fernandez-Cabezudo M.J., Al-Ramadi B.K. (2021). The Immunomodulatory Effects of Honey and Associated Flavonoids in Cancer. Nutrients.

[43] Al-Waili N.S., Boni N.S. (2003). Natural honey lowers plasma prostaglandin concentrations in normal individuals. J. Med. Food.

[44] Santos L.M., Fonseca M.S., Sokolonski A.R., Deegan K.R., Araújo R.P., Umsza-Guez M.A., Barbosa J.D., Portela R.D., Machado B.A. (2020). Propolis: Types, composition, biological activities, and veterinary product patent prospecting. J. Sci. Food Agric..

[45] Ansorge S., Reinhold D., Lendeckel U. (2003). Propolis and some of its constituents down-regulate DNA synthesis and inflammatory cytokine production but induce TGF-beta1 production of human immune cells. Z. Naturforsch C J. Biosci..

[46] Almasaudi S.B., El-Shitany N.A., Abbas A.T., Abdel-dayem U.A., Ali S.S., Al Jaouni S.K., Harakeh S. (2016). Antioxidant, Anti-inflammatory, and Antiulcer Potential of Manuka Honey against Gastric Ulcer in Rats. Oxid. Med. Cell Longev..

[47] Gomes T., Feás X., Iglesias A., Estevinho L.M. (2011). Study of organic honey from the Northeast Portugal. Molecules.

[48] Schley P.D., Field C.J. (2002). The immune-enhancing effects of dietary fibres and prebiotics. Br. J. Nutr..

[49] Sanz M.L., Polemis N., Morales V., Corzo N., Drakoularakou A., Gibson G.R., Rastall R.A. (2005). In vitro investigation into the potential prebiotic activity of honey oligosaccharides. J. Agric. Food Chem..

[50] Brudzynski K., Miotto D., Kim L., Sjaarda C., Maldonado-Alvarez L., Fukś H. (2017). Active macromolecules of honey form colloidal particles essential for honey antibacterial activity and hydrogen peroxide production. Sci. Rep..

